# Validation of doubled haploid plants by enzymatic mismatch cleavage

**DOI:** 10.1186/1746-4811-9-43

**Published:** 2013-11-13

**Authors:** Bernhard J Hofinger, Owen A Huynh, Joanna Jankowicz-Cieslak, Andrea Müller, Ingrid Otto, Jochen Kumlehn, Bradley J Till

**Affiliations:** 1 Plant Breeding and Genetics Laboratory, Joint FAO/IAEA Division, International Atomic Energy Agency, Vienna International Centre, PO Box 100, A-1400, Vienna, Austria; 2 Leibniz Institute of Plant Genetics and Crop Plant Research (IPK) Gatersleben, Plant Reproductive Biology, Corrensstrasse 3, D-06466 Seeland, OT Gatersleben, Germany

**Keywords:** Polymorphism discovery, TILLING, Single-strand-specific nuclease, Loss of heterozygosity

## Abstract

**Background:**

Doubled haploidy is a fundamental tool in plant breeding as it provides the fastest way to generate populations of meiotic recombinants in a genetically fixed state. A wide range of methods has been developed to produce doubled haploid (DH) plants and recent advances promise efficient DH production in otherwise recalcitrant species. Since the cellular origin of the plants produced is not always certain, rapid screening techniques are needed to validate that the produced individuals are indeed homozygous and genetically distinct from each other. Ideal methods are easily implemented across species and in crops where whole genome sequence and marker resources are limited.

**Results:**

We have adapted enzymatic mismatch cleavage techniques commonly used for TILLING (Targeting Induced Local Lesions IN Genomes) for the evaluation of heterozygosity in parental, F1 and putative DH plants. We used barley as a model crop and tested 26 amplicons previously developed for TILLING. Experiments were performed using self-extracted single-strand-specific nuclease and standard native agarose gels. Eleven of the twenty-six tested primers allowed unambiguous assignment of heterozygosity in material from F1 crosses and loss of heterozygosity in the DH plants. Through parallel testing of previously developed Simple Sequence Repeat (SSR) markers, we show that 3/32 SSR markers were suitable for screening. This suggests that enzymatic mismatch cleavage approaches can be more efficient than SSR based screening, even in species with well-developed markers.

**Conclusions:**

Enzymatic mismatch cleavage has been applied for mutation discovery in many plant species, including those with little or no available genomic DNA sequence information. Here, we show that the same methods provide an efficient system to screen for the production of DH material without the need of specialized equipment. This gene target based approach further allows discovery of novel nucleotide polymorphisms in candidate genes in the parental lines.

## Background

The first report of haploid production in plants dates to the early 20^th^ century [1]. The approach is especially powerful in crop breeding because haploid plants are in many cases easily made diploid either spontaneously or *via* treatment with chemicals such as colchicine. This allows the generation and rapid fixation of genetic variants in a homozygous state. The result is true breeding material with fixed traits that can be used for a variety of approaches in research and crop improvement. The broad utility of this approach is highlighted by the development of DH methods for more than 250 species [2]. A wide range of techniques have been described for DH production including pollen embryogenesis, gynogenesis and uniparental genome elimination upon interspecific crosses or pollination by inducer lines [3]. In barley (*Hordeum vulgare* L.), the so-called *bulbosum*-technique initially enjoyed a fairly broad application. It relies upon crossings between the two species followed by the spontaneous elimination of the *H. bulbosum* chromosomes, which entails the formation of *H. vulgare* haploids [4,5]. Later, however, more efficient and reliable protocols were developed for the production of DHs from immature barley pollen cultivated while either still inside dissected anthers or after isolation [6-8]. These methods are not only standard in current barley breeding programs, but have also been essential for basic research on pollen embryogenesis [9,10], the development of unique transformation technology and its use in applied research and biotechnology [11-14]. Yet, for all the efforts to produce DHs, efficiencies can vary dramatically for largely unknown reasons and many species and genotypes remain recalcitrant. New approaches may help to address this problem. For example, doubled haploidy could be induced in *Arabidopsis thaliana* by modification of the centromere-specific histone CENH3 [15]. In theory, this approach could be applied to other species allowing great improvements in plant breeding.

While promising, production of DHs does not occur 100% of the time. The rate of production can vary depending on species and method used. For example, haploid inducing lines in maize show rates of 8 to 10% of haploid embryo formation, while all other individuals obtained are unwanted products of regular fertilization events [16]. Moreover, plants can derive from maternal tissue rather than from gametophytic (haploid) cells in anther and ovule/ovary culture [17]. Validation that produced plants are truly haploid or DH is therefore necessary to avoid unwanted heterozygosity in downstream applications. A thorough analysis of putative haploids is especially important when novel methods are developed and when methods are transferred to different laboratories. Without validation, unforeseen decreases in the efficiency of DH production can add years of extra effort to research and breeding projects. We sought to evaluate molecular screening methods to test for homozygosity that are applicable across most crop species. Enzymatic mismatch cleavage methods for polymorphism discovery in heteroduplexed DNA molecules have been widely applied in TILLING reverse-genetics projects [18,19]. A standard approach includes PCR amplification of ~1 to 1.5 kb gene targets followed by denaturation and annealing to form heteroduplexed molecules that are single stranded where nucleotide polymorphisms exist. This is followed by incubation with a single-strand-specific nuclease that cleaves the DNA at the site of mismatch. The approach is advantageous because with only minor modifications it can be applied to most species. Further, it has proven to be a highly accurate and robust approach for polymorphism discovery in both diploid and polyploid species [20-23]. A range of enzymes has been described for mismatch cleavage including mung bean nuclease, extracts from brassica petioles, and crude extractions from celery [24,25]. Double strand breaks can be induced with these enzymes, allowing for low-cost native agarose gels to be used as a readout platform [26,27]. When individuals are screened alone using these methods, only heterozygous polymorphisms are detected. If samples are mixed, both homozygous and heterozygous variation can be discovered, allowing unambiguous assignment of polymorphisms. When used to evaluate natural nucleotide polymorphisms, this approach has been termed EcoTILLING [28]. Thus nucleotide diversity and the loss of heterozygosity can be easily evaluated in hundreds of individuals without the need for DNA sequencing [20]. The aim of this study was to adapt these methods for rapid evaluation of loss of heterozygosity in specific gene sequences in putatively DH plants. Microsatellite marker methods have been previously used to screen for heterozygosity in a non gene-specific manner in a variety of plants including barley, *Brassica, Mimulus*, oil palm and potato [29-33]. We used SSR markers, which are well established in barley, as a baseline to compare the efficiency of enzymatic mismatch cleavage for validating DH material.

## Results and discussion

### Development of polymorphic markers for loss of heterozygosity screens

**Figure 1:**
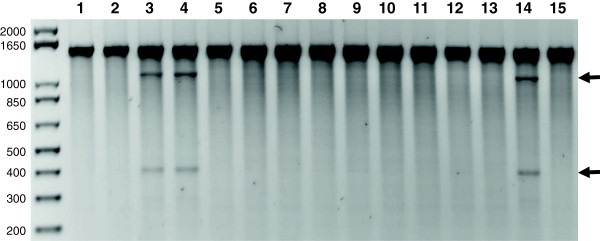
**Agarose gel evaluation of doubled haploid production in barley by enzymatic mismatch cleavage.** Enzymatic mismatch cleavage was carried out to evaluate homozygosity in putative barley doubled haploid lines. A 1476 bp fragment of the barley *Mlo9* gene was PCR amplified and digested with a crude celery juice extract (CJE) containing single-strand-specific nuclease activity followed by agarose gel analysis. The top band in lanes 1-15 represents undigested PCR product. The cleavage products present in heterozygous samples are marked with arrows. Parental lines Golden Promise (GP) and HOR1606 are homozygous for this gene region (lanes 1 and 2 respectively). A synthetic mixture of parental DNA and also the F1 sample from crossing of the two parents show cleavage fragments resulting from a heterozygous SNP (lanes 3 & 4). Doubled haploid plants (lanes 5-13) are homozygous. Mixtures of genomic DNA from a DH plant and GP show cleavage products while mixture of the same material with HOR1606 does not, indicating the DH harbors the GP allele (lanes 14 & 15).

**Figure 2:**
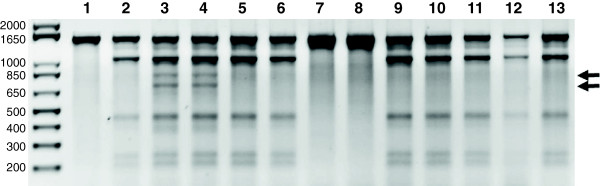
**Doubled haploid screening and polymorphism discovery in the *nbs2-rdg2a *locus.** A 1500 bp gene fragment of the barley *nbs2-rdg2a* locus was PCR amplified and subjected to enzymatic mismatch cleavage. Parental line Golden Promise is homozygous in the gene region while HOR1606 shows banding indicative of co-amplification of homologous gene copies (lanes 1 and 2 respectively). Heterozygous polymorphisms marked by arrows are found in a synthetic mixture of parental genotypes and in DNA from an F1 hybrid of the cross of the two parents (lanes 3 & 4). Doubled haploids in lanes 5-13 are all homozygous. Banding patterns indicate if the gene target originates from GP or HOR1606.

To evaluate the practical use of an enzymatic mismatch cleavage approach for the screening of homozygosity in putative DH lines of barley, we carried out a pilot experiment to determine the presence of nucleotide polymorphisms in parental plants. We tested the parental lines Golden Promise and HOR1606 and synthetic mixtures of the two parental genomic DNAs with a total of 26 primer pairs that were previously developed for different barley TILLING projects (Table 1). Primer pairs that allow the detection of novel heterozygosity in F1 hybrids not present in the parental lines are considered suitable for DH screening. Five of the tested primer pairs did not amplify a PCR product in at least one of the two parents and were therefore excluded from further experiments (Table 2). The remaining primer pairs produced full-length PCR products in both parents, with four producing substantially lower yields. Consequently, nuclease digestions of these amplicons using a crude celery juice extract (CJE) yielded low concentration cleavage products making visual gel analysis difficult. One primer pair produced a high yield of PCR product, but weak cleavage product banding. Five primer pairs produced a high yield of PCR product but no detectible enzymatic cleavage. The remaining eleven primer pairs produced both high yielding PCR product and high concentration cleaved bands when treated with nuclease, and were thus deemed suitable for DH screening (Figures 1 and 2 and Additional file 1). In addition, cleavage products in synthetic mixtures of genomic DNA of the two parents prior to PCR were observed indicating homozygous polymorphisms between the parents (for example, Figure 1). This resulted in the validation of 11/26 primer pairs suitable for DH screening.

**Table 1:** Primer sequences, PCR product sizes, and references for enzymatic mismatch cleavage

**Number**	**Primer name**	**Primer sequence**	**Product size (bp)**	**Source**
1	rdg2a_F1	CTTGCTCTCAAGACAATGGGTGGATTG	1499	1
	rdg2a_R1	TCCAAACTGCTAAACATCCGAGGCTCT		
2	rdg2a_F2	CTCTCAAGACAATGGGTGGATTGCTGA	1493	1
	rdg2a_R2	CAAACTGCTAAACATCCGAGGCTCTCC		
3	rdg2a_F3	TCGCTATGTCAAGAGCTGGATGAAGGA	1496	1
	rdg2a_R3	AAGTGCGTAGGATTGTTCTGCCTTTGC		
4	nbs2-rdg2a_F1	GCTCTTCCGTTTTGAAATGAGCAGGAA	1503	1
	nbs2-rdg2a_R1	TGTTTTGCATTTATGGCCTTGCAAATG		
5	nbs2-rdg2a_F2	TCCACTACCCGAAAGGCACTCAGCTAC	1500	1
	nb2-rdg2a_R2	GCAATGCAATGCTCTTACTGACGCAAA		
6	nb2-rdg2a_F3	TCGAACGAATCAGTGGGTTATGCAAAG	1497	1
	nb2-rdg2a_R3	ATGAAGTGTTCCCCTCCAGGTTGTCAC		
7	nbs3-rdg2a_F1	TGGCAAGTCCACTACCAAAAAGGCACT	1491	1
	nbs3-rdg2a_R1	GCTCTTAGTGATGCCAATACCCGTTGC		
8	nbs3-rdg2a_F2	GCTCTTCCGTTTTGAAATGAGCAGGAA	1491	1
	nbs3-rdg2a_R2	TGTTTTGCATTTATGGCCTTGCAAATG		
9	HVgna1f	GACCCAGATGGCATCCAC	552	2
	HVgna1r	ATGCGACGAGACAAAGGAAT		
10	HVraa1f	GTCGACGACTTGCATCATCTATCG	545	2
	HVraa1R	CACCCCGATCACTAACACACAA		
11	HV_hpa1F	CCCTTATGTGTACCCTGATCCTGA	1100	2
	HV_hpa1R	GGTCCAACAGACGTATTAGCCAAG		
12	HV_Mlo9-F1	AGCAAACCAGACACACAGCAGCGTACC	900	3
	HV_Mlo9-R1	GCAAAGGCTCACTTTGAGACGGCTTAG		
13	HV_Mlo9-F2	CATTTGTCGCAAAACAGCAAGTTCGAC	1476	3
	HV_Mlo9-R2	TTGTCTCATCCCTGGCTGAAGGAAAAA		
14	HvHox1-F1	AAGCATGGACAAGCATCAGCTCTTTGA	985	3
	HvHox1-R1	GGCAGCAGCTATCTCGGCTATTTTATGG		
15	HV_Kap1_c1_F	TCCACCGGTAAAGAAACCAG	1030	4
	HV_Kap1_c1_R	TGAGGGAGGGAGAAAGATGA		
16	HV_Kap1_d1_F	CTCCCTCCCTCAAGAAATCC	899	4
	HV_Kap1_d1_R	GCTGTCGCAAAATACAGCAA		
17	HV_Kap1_e1_F	TTGCTGTATTTTGCGACAGC	1136	4
	HV_Kap1_e1_R	CATGTGTTAAAAGCCGCAGA		
18	HV_Kap1_g1_F	ATTGAGTGCCTCTCGGCTTA	1175	4
	HV_Kap1_g1_R	TGAGGAAAGAAGGGATGTGG		
19	HV_Kap1_h1_F	CCACATCCCTTCTTTCCTCA	718	4
	HV_Kap1_h1_R	GGGAGCTTGCCTTTCTTCTT		
20	HV_Kap1_i1_F	TGTGGAACTATAAATCTGGCTTCA	698	4
	HV_Kap1_i1_R	CGAGCTAGCCGAACCTGTAG		
21	Hv_mloA-F	CGTGTGCGTACCTGGTAGAG	599	5
	Hv_mloA-R	CAAGCCAAGACGACAATCAG		
22	Hv_mloB-F	CTGATTGTCGTCTTGGCTTG	624	5
	Hv_mloB-R	CTGACTCCATACGCCAAACA		
23	Hv_mloC-F	TGTTTGGCGTATGGAGTCAG	566	5
	Hv_mloC-R	AGAAACCGGAGAGGAGAAGG		
24	Hv_mloD-F	CCTCACCCTCTTCCTTGACA	574	5
	Hv_mloD-R	CGTCAGAGCAGTTCATCAGC		
25	Hv_mloE-F	CCACCGATGAACTTGTCAGT	837	5
	Hv_mloE-R	GAGAGGGGTTTTGTTTGTGC		
26	Hv_Mla1-3 F	AGCAGCTCGACAGCCAAGACAA	451	5
	Hv_Mla1-3R	CCCAACCCTCCAAATCCAACAA		

^1^J. Jankowicz-Cieslak and B.Till (unpublished).

^2^[34].

^3^[35].

^4^B.Till and F. Taassob Shirazi (unpublished).

^5^[36].

**Table 2:** Summary of TILLING primers tested, quality of PCR products, CJE digestion products and polymorphisms detected

**Primer pair**	**PCR product in the parental lines**		**CJE digestion**		**Polymorphisms detected between parental lines**	**Tested in DH material**
	**GP**^**1**^	**HOR1606**	**GP**^**2**^	**HOR 1606**	**GP + HOR1606**	
rdg2a_F1 + R1	N	Y	---	---	---	N
rdg2a_F2 + R2	N	Y	---	---	---	N
rdg2a_F3 + R3	N	W	---	---	---	N
nbs2-rdg2a_F1 + R1	W	W	?	?	N	N
nbs2-rdg2a_F2 + R2	Y	Y	F	C	Y	Y
nbs2-rdg2a_F3 + R3	W	W	?	?	N	N
nbs3-rdg2a_F1 + R1	Y	Y	F	C	Y	Y
nbs3-rdg2a_F2 + R2	N	N	---	---	---	N
HVgna1f + r	N	N	---	---	---	N
HVraa1f + R	W	W	?	?	N	N
HV_hpa1F + R	W	W	?	?	N	N
HV_Mlo9-F1 + R1	Y	Y	F	F	Y	Y
HV_Mlo9-F2 + R2	Y	Y	F	F	Y	Y
HvHox1-F1 + R1	Y	Y	F	F	N	N
HV_Kap1_c1_f + r	Y	Y	F	F	Y	Y
HV_Kap1_d1_f + r	Y	Y	F	F	Y	Y
HV_Kap1_e1_f + r	Y	Y	F	F	Y	Y
HV_Kap1_g1_f + r	Y	Y	F	F	N	N
HV_Kap1_h1_f + r	Y	Y	F	F	N	N
HV_Kap1_i1_f + r	Y	Y	F	F	N	N
Hv_mloA-F + R	---	---	---	---	Y	Y
Hv_mloB-F + R	---	---	---	---	Y	Y
Hv_mloC-F + R	---	---	---	---	Y	Y
Hv_mloD-F + R	---	---	---	---	Y	Y
Hv_mloE-F + R	---	---	---	---	N	N
Hv_Mla1-3 F + 3R	---	---	---	---	N	N

^**1**^Y = yes, W = weak amplification, N = no.

^2^F = full-length PCR product, C = cleavage products present, ? = unclear results, --- = not tested.

In order to compare the efficiency of the enzymatic mismatch cleavage approach with an existing method based on SSR markers, we tested 32 previously published barley markers. Useful SSR markers are defined as those that amplify a different molecular weight band in each parental material. This allows a clear differentiation between progeny from doubled haploidy versus those maintaining both alleles from the F1 parent. Table 3 summarizes the results of the pilot experiments with the parental lines Golden Promise and HOR1606. Eight primer pairs did not amplify a PCR band in at least one of the two parents. One primer pair could not be used because it amplified several bands. Thirteen primer pairs amplified PCR products in both parents, but no molecular weight polymorphism could be detected between the two parents (Additional file 2). Seven SSR markers did not give a clear result because of limited separation capability of the agarose gels. Although a relatively short length polymorphism cannot be excluded for these markers, they were deemed not suitable for an agarose gel-based screening approach (Additional file 2). Only three of the primer pairs tested showed a clear length polymorphism of the PCR products between Golden Promise and HOR1606 after separation on standard agarose gels, making them candidates for the DH screening experiments.

**Table 3:** Summary of SSR primers tested, quality of PCR products and polymorphisms detected

**Primer pair**^**1**^	**PCR amplification**^**2**^		**Polymorphisms detected between parents**
	**GP**	**HOR1606**	**GP + HOR1606**
cnl34	W	W	N
cnl73	?	?	N
cnl31	Y	Y	N
cnl146	Y	Y	Y
cnl130	Y	Y	?
cnl151	N	Y	---
cnl140	N	N	---
HVACL1	Y	Y	?
HVLEU	Y	Y	N
HVGNIRE	Y	Y	N
HVCSG	N	N	---
HVADH1	N	N	---
HVWAXYG	?	?	N
HVDHN7	Y	?	?
HVCMA	Y	Y	Y
HVDHN9	Y	Y	N
HVBKASI	Y	W	?
HVPRP1B	?	Y	?
HVBDG	W	W	N
HVBARE1	N	N	---
HVRCABG	W	N	---
HVSIP1A	Y	Y	N
Bmac0113	Y	Y	Y
Bmag0323	Y	Y	N
Bmac0812	Y	Y	N
Bmag0477	W	W	?
Bmag0387	Y	W	?
Bmac0163	Y	Y	N
GBM1284	?	?	---
Bmag0346	N	N	---
Bmag0023	N	N	---
GBM1300	Y	Y	N

^1^From http://germinate.scri.ac.uk/ssr/barley_s.html.

^2^Y = yes, W = weak amplification, N = no, ? = unclear results, --- = not tested.

In summary, the pilot experiments showed that 11 out of 26 (42.3%) primer pairs are suitable for screening of loss of heterozygosity in DHs using a single-strand-specific nuclease approach. The pilot experiments of the SSR markers revealed only 3 out of 32 (9.4%) primer pairs were suitable for screening.

### Evaluation of homozygosity in DH plants using an enzymatic mismatch cleavage approach

Based on the pilot experiments, we selected a 1476 bp gene target of the powdery mildew resistance locus *Mlo9* to test the applicability of enzymatic mismatch cleavage for evaluation of homozygosity in putative DH plants of barley [35] (Figure 1). After treatment of the PCR amplification products using CJE, both parental lines, Golden Promise and HOR1606, showed the 1476 bp full-length PCR product indicating homozygosity in the amplified region. In a synthetic mixture of the two parental genomic DNAs before PCR and in F1 plants derived from crossings of the two parents, two cleavage products of approximately 400 bp and 1100 bp were present. These results can be explained by a single-nucleotide polymorphism between the two parents; the molecular weights of the two cleaved fragments sum to the molecular weight of the full-length amplicon. The single-nucleotide polymorphism between the two parental lines was verified by Sanger sequencing (data not shown). All nine DH plants that were produced for this study from the heterozygous F1 generation (see Methods) showed only the 1476 bp full length PCR product. The absence of cleavage bands demonstrated that all of the tested plants are homozygous. To determine the parental origin of the DHs, genomic DNA of the DH material was mixed in equal concentration with DNA from the parental lines prior to PCR and enzymatic digestion. Figure 1 shows an example where mixture with the parental line Golden Promise produced the two mismatch cleavage products. Therefore the genotype of this DH plant in the tested region is different from Golden Promise. As a cross check, a mixture of the same DH plant with the other parent HOR1606 showed the full-length PCR product but no cleavage products, verifying that this DH plant carries the HOR1606 allele. Similar data was produced with other primer pairs (Additional file 1).

Determination of the parental origin of the DH material also serves as a positive control for enzymatic mismatch cleavage of the heterozygous sites previously determined in F1 material. Each DH individual is assigned to either one or the other (but not both) parental genotypes through the production of cleaved PCR product in the assay. A master mix of CJE is used and applied to all samples and all samples are incubated simultaneously. Thus screening assays contain internal positive and negative controls for enzymatic mismatch cleavage. Previous studies using enzymatic mismatch cleavage for discovery of rare polymorphisms in polyploids and diploids showed false positive and false negative error rates at or below 6% [20,37]. The DH screening procedure presented here is unique to previous work in two important ways. Amplicons are first selected based on discovery of heterozygous polymorphisms. Amplicons where no polymorphisms are detected are not used for screening (Additional file 1). Thus false negative discovery errors are removed before genotyping of the DHs begins. Secondly, the presence of both positive and negative controls in the genotyping assay ensures that enzymatic cleavage activity can be monitored and false positive errors can be detected.

In addition to errors in genotyping, it is also important to consider the chance of misassignment of a plant as being homozygous when it is actually heterozygous. The expected frequencies of not producing a true DH plant will vary depending on the method used for production of DHs and the relative skill of the researcher. The appropriate number of gene targets and samples should therefore be screened to control for this. As an example, probabilities can be calculated for assigning a plant as DH when it is in reality the product of an accidental self-fertilization event. Assuming a normal segregation ratio of 1:2:1, there is a probability of 0.5 that a heterozygous plant is misassigned as homozygous, or DH. When screening 4 unlinked loci, this drops to 0.5^4^ (0.0625). This may be a reasonable confidence for many projects. Increasing the number of target loci to 10 gives a 99.9% chance that the plant is truly homozygous. This should be suitable for even the most valuable material. If segregation bias is a concern, amplicons can be chosen in regions of the genome such as introns where selective pressure is reduced. Indeed, this strategy should result in an increased number of discoverable heterozygous polymorphisms and therefore improve the efficiency of the method. It should be noted that methods for differentiating heterozygous versus homozygous plants are by no means suitable to differentiate haploid versus DH plants. In many crops, this is easily determined phenotypically, e.g. by plant vigour or fertility, while flow cytometry allows for an accurate determination of ploidy even at a very early plant developmental stage.

### Evaluation of a more complex target gene for DH screening by enzymatic mismatch cleavage

In the present study, we discovered two primer pairs that produced a more complex cleavage pattern. Parental line Golden Promise showed the full-length PCR product indicating homozygosity in this gene region, while parent HOR1606 showed cleavage products on the agarose gel in the *nbs2-rdg2a* and *nbs3-rdg2a* primer pairs (Figure 2, and Additional file 1B). Sequencing of the PCR product of HOR1606 did not provide any evidence for heterozygosity, but suggested the presence of three very similar gene copies in this genetic background (data not shown). Indeed, positional cloning of the *Rdg2a* locus showed three genes whose open reading frames were 87-90% identical at the DNA level [38]. Thus, the observed complex cleavage pattern is most likely a result of co-amplification of the gene copies during PCR. Cleavage products are observed if nucleotide polymorphisms exist between copies. This phenomenon has been reported in soybean [39]. Further, in polyploid banana, co-amplification of homeologous sequences was exploited to evaluate natural nucleotide variation in diverse accessions where the method showed greater than 95% accuracy in detecting polymorphisms in PCR amplicons [20]. This approach was further exploited to discover EMS induced mutations in TILLING screens [21]. Therefore, the presence of cleavage products in the parental barley line is not expected to adversely affect the ability to screen for loss of heterozygosity. In a synthetic mixture of the two barley parents and in F1 plants derived from crossings between the two parents, all the cleavage bands of HOR1606 were present when screening with primer pair *nbs2-rdg2a*, but also two additional bands of ~ 800 bp and 700 bp (Figure 2). This additional set of cleavage products indicates at least one single nucleotide polymorphism between the two parental lines. Two of the DH plants examined showed the full-length product like Golden Promise, the other seven DH plants showed the cleavage pattern of HOR1606. This banding pattern indicates the presence of either of the two parental alleles in each DH plant. The absence of the additional bands at ~ 800 bp and 700 bp indicates that all of these plants were homozygous in the region tested. While the banding pattern is more complicated, a cleavage pattern in one of the parents has the advantage that it can be used to track inheritance of the parental genotypes in the DH material without the need of synthetic mixtures with the parental lines (also see Additional file 1B).

### Evaluation of homozygosity in DH plants using SSR markers

**Figure 3:**
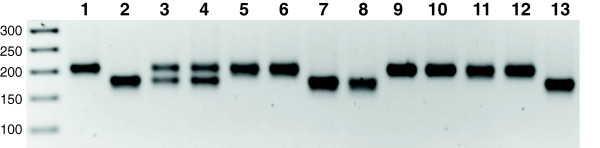
**Agarose gel image of evaluation of doubled haploid production in barley using a SSR marker.** SSR marker Bmac0113 displays molecular weight polymorphism in amplified bands between parental lines Golden Promise (GP) and HOR1606 (lanes 1 and 2 respectively). Amplification of GP produces a band of approximately 210 bp and HOR1606 produces a band of approximately 175 bp. A synthetic mixture of parental genomic DNA and F1 material from crossing of the two parents shows both bands (lanes 4 & 5). Doubled haploid plants produce either parental band (lanes 5-13).

We next examined microsatellite marker Bmac0113 located on chromosome 5 to test DH screening using the SSR approach (Figure 3). The two parental lines Golden Promise and HOR1606 produced each a single PCR band indicating the homozygous state of the marker region. However, there is a clear variation in the size of the two PCR products: approximately 175 bp in Golden Promise and 210 bp in HOR1606. A synthetic mixture of the two parental DNAs and the F1 generation derived from crossings of the two parents showed the presence of both bands at similar intensities indicating heterozygosity. Each of the nine DH plants, which were produced from the F1 generation, showed only a single band verifying the return to a homozygous state in these plants. Six of the DHs inherited the genotype of parent Golden Promise and three of them that of parent HOR1606.

## Conclusions

In this study we developed a low-cost approach utilizing self-extracted enzyme, PCR, and standard agarose gel electrophoresis to evaluate the production of DHs in barley. Twenty-six primer pairs were needed to identify eleven that were suitable for enzymatic mismatch cleavage screening. For SSR markers, 32 primer pairs were screened in order to identify 3 that were polymorphic in the F1 material. Considering that both methods utilize unlabelled oligonucleotide primers and Taq polymerase, assay costs are roughly comparable. Self-extracted CJE costs less than one cent per reaction. Thus costs are lower in the enzymatic mismatch cleavage approach. The frequency of identifiable heterozygous SNPs in parental material will vary depending on genetic background and target amplicon choice, as will the frequency of suitable SSR primers. Therefore, costs and efficiencies of the enzymatic cleavage method are expected to vary. However, a further advantage of enzymatic mismatch cleavage is that the same methods can be applied to most diploid and polyploid species, even where limited DNA sequence information is available. The only requisite is PCR amplification of an appropriately sized amplicon and the presence of polymorphisms between parental materials. Data from this comes from many TILLING projects in different species where only minor modifications of the input genomic DNA concentration for PCR allowed efficient enzymatic mismatch cleavage for the discovery of rare induced mutations [18]. Therefore, we expect enzymatic mismatch cleavage for DH screening to remain efficient in lesser studied crops that lack well developed marker systems. Further, novel genetic variation between parental lines in specific gene targets of interests can be discovered during the screening process. This gene sequence based approach allows selection of DH plants that are genetically distinct from each other while avoiding unnecessary replication of potentially clonally related material. Additionally, discovery of novel genetic variation in parental lines can provide new insights into gene function. We envision other applications using these methodologies where rapid and low-cost methods for evaluating loss of heterozygosity are sought.

## Methods

### Production of DH lines

Barley cv. ‘Golden Promise’ that produces light colored grains, and ‘Weihenstephaner Schwarze Nackte’ (Gatersleben Genbank accession HOR1606) were used as parental lines. For the production of F1 grains, spikes of the female parent ‘Golden Promise’ were emasculated 2 to 3 days prior to anthesis by removal of the anthers using fine-tipped forceps. Any unintended pollination was prevented by isolating the emasculated spikes in polyethylene bags. When the stigmas were fully receptive, anthers containing mature pollen from the male parent HOR1606 were collected in a plastic petri dish, and then 1 or 2 anthers placed on top of the pistil in each emasculated floret. The bags were kept on the spikes for another week and then removed to avoid fungal contaminations.

F1 hybrids were grown from April to July under field-like conditions in partially open, small greenhouses at the campus of IPK Gatersleben. DH lines were produced via pollen embryogenesis. To this end, the spikes were harvested when the most pollen grains were at a stage just prior to mitosis I. Isolation and culture of immature pollen was conducted largely following a method previously described [7]. Specifically, pollen embryogenesis was triggered by spike pretreatment at 4°C for three weeks followed by starvation treatment of isolated pollen in SMB medium at 25°C for two days, before the pollen was transferred to rich (KBP) medium. To prevent bacterial contamination, the media were supplemented with 100 mg/L Cefotaxime and 150 mg/L Timentin.

A total of 10 regenerated plants were established in soil; one was haploid and nine were diploid, as measured by a Ploidy Analyser I (Partec, Münster, Germany) following the manufacturer’s instructions. Pure segregation of a single grain color per progeny clearly indicated entire homozygosity of all lines generated. Nine putatively DH lines were used for molecular analysis.

### Plant material and growth conditions

Grains from selfed and crossed parental lines, F1 plants and DH lines as specified above were germinated on wet filter paper and after germination transferred to pots and grown in a greenhouse until the three leaf stage. Barley leaf material was harvested between three to four weeks after planting. One hundred mg of leaf material was collected in 2 mL tubes and quick-frozen in liquid nitrogen and either immediately used for DNA isolation or stored at -80°C until DNA isolation.

### Isolation of genomic DNA

Three tungsten carbide beads of 3 mm diameter (Qiagen, Valencia, CA, USA) were added to 2 mL tubes containing the leaf samples. Leaves were ground in liquid nitrogen 2 times using a Qiagen TissueLyser II (10 sec at 1/30 frequency). DNA isolation was carried out with Qiagen DNeasy Plant Mini Kit according to kit instructions or with slightly modified incubation times to increase DNA yield. DNA yield was measured on a NanoDrop 1000 Spectrophotometer (Thermo Scientific, Wilmington, DE, USA) and DNA quality was assessed by gel electrophoresis.

### Heteroduplex mismatch cleavage screening

PCR reactions for enzymatic cleavage were carried out in 25 μL volumes under the following conditions (per reaction): 1x Ex Taq™ Reaction Buffer (TaKaRa, Shiga, Japan), 0.2 mM TaKaRa dNTP mixture, 0.5 U TaKaRa Ex Taq™ Polymerase, 0.12 μM of forward and reverse primer and 25 ng genomic DNA. The target genes and sequences of the 26 TILLING primer pairs tested are summarized in Table 1. Design of new primers and PCR amplification was carried out as previously described [19], except that annealing temperatures were adjusted according to the melting temperature of the primer. An annealing temperature of 65°C for primer pairs 1-14, 55°C for primers 15-20 and 60°C for 21-26 was used (Table 1). A 5 μL aliquot of the undigested PCR products was separated by agarose gel electrophoresis to check the performance of the PCR. Ten μL aliquots of the PCR products were used for enzymatic mismatch cleavage using a crude CJE containing the nuclease CELI. CJE and reaction buffer were prepared as previously described [19]. Digestion reactions were as follows: 10 μl PCR product, 5 μL dH_2_O, 1.5 μL celery juice extract (CJE) buffer and 3.5 μL CJE were mixed and incubated for 15 min at 45°C. The digestions were stopped by cooling the reaction to 8°C and adding 55.56 mM (final concentration) EDTA (pH = 8.0). Ten μL of each reaction were separated by gel electrophoresis on a 1.5% agarose gel stained with ethidium bromide.

### SSR analysis

Barley SSR primer sequences were taken from Barley SSRs 1.0 (http://germinate.scri.ac.uk/ssr/barley_s.html). PCR reactions for SSR marker amplification were carried out in 25 μL volumes under the following conditions (per reaction): 1x Taq buffer without MgCl_2_ (Roche Applied Science, Mannheim, Germany), 1.5 mM MgCl_2_, 0.4 mM PCR nucleotide mix (Promega, Madison, WI, USA), 1.25 U aTaq DNA polymerase (Promega, Madison, WI, USA), 0.32 μM of forward and reverse primer and 20 ng genomic DNA. The PCR conditions for SSR marker amplifications were: 5 min at 94°C; 35 cycles (1 min at 94°C; 1 min annealing either at 55°C or 58°C depending on the SSR marker; 30 sec at 72°C); a final elongation at 72°C for 5 min. Aliquots of the PCR products were separated by 2% agarose gel electrophoresis and visualized by staining with ethidium bromide.

## Authors’ contributions

JK and BJT conceived of the study. JK, AM and IO participated in the design, execution, data collection and critical analysis of the production of DH plants. BJH, OAH, JJC and BJT participated in the design, execution, data collection and critical analysis of the enzymatic mismatch cleavage portion of this work. BJH and BJT prepared the manuscript, and JK, OAH and JJC revised it critically. All authors read and approved the final manuscript.

## Supplementary Material

Additional file 1 **Agarose gel images of primers evaluated for DH screening by enzymatic mismatch cleavage.** Screening with primer pairs HV_Mlo9 (A), and nbs3_rdg2a (B), resulted in the production of cleaved products (marked by arrow heads) in mixed parental and F1 DNA samples indicating suitability for screening doubled haploid plants (lanes marked DH). Primers for nbs3_rdg2a, like those for nbs_rdg2a (Figure 2) produce banding in HOR1606 samples (marked by *), allowing assignment of parental origin in doubled haploid plants without the need to mix DNA from parental genotypes. Primer pairs with a melting temperature of 60°C are also suitable for DH screening (C, D, E). Rapid testing of primer pairs is accomplished by screening by comparing untreated PCR products with those treated with CJE (F). Primer pairs where amplification is observed but no polymorphisms detected in mixed parental or F1 DNA samples are not used for screening putatively doubled haploid material (G, and not shown). Click here for file

Additional file 2 **Agarose gel images of SSR markers for doubled haploid screening.** Pilot tests were performed to identify primers showing polymorphism between parental Golden Promise (GP) and HOR1606 lines. No DNA controls and an additional parent, HOR2444, were included in each trial. Primer names are included below the image. Examples include weakly amplifying primers (cnl34), primers where no polymorphisms are observed (cnl73, cnl31, cnl130), where mis-amplification occurred in one of the parents (cnl151) and where a size polymorphism could be detected between GP and HOR1606 (cnl146). Further optimization was performed with cnl146 by reducing extension time from 2 minutes to 30 seconds (not shown). Click here for file
