# Supplementary material for: Validation of doubled haploid plants by enzymatic mismatch cleavage
Source: Plant Methods. 2013 Nov 13;9:43. doi: 10.1186/1746-4811-9-43 (PMC3831592; doi:10.1186/1746-4811-9-43)

Additional file 1: Agarose gel images of primers evaluated for DH screening by enzymatic mismatch cleavage. Screening with primer pairs HV_Mlo9 (A), and nbs3_rdg2a (B), resulted in the production of cleaved products (marked by arrow heads) in mixed parental and F1 DNA samples indicating suitability for screening doubled haploid plants (lanes marked DH). Primers for nbs3_rdg2a, like those for nbs_rdg2a (figure 2) produce banding in HOR 1606 samples (marked by *), allowing assignment of parental origin in doubled haploid plants without the need to mix in DNA from parental genoytpes. Primer pairs with a melting temperature of 60⁰C are also suitable for DH screening (C,D, E). Rapid testing of primer pairs is accomplished by screening by comparing untreated PCR products with those treated with CJE (F). Primer pairs where amplification is observed but no polymorphisms detected in mixed parental or F1 DNA samples are not used for screening putatively doubled haploid material (G, and not shown).

A.

Primer pair: HV_Mlo9_F1_R1


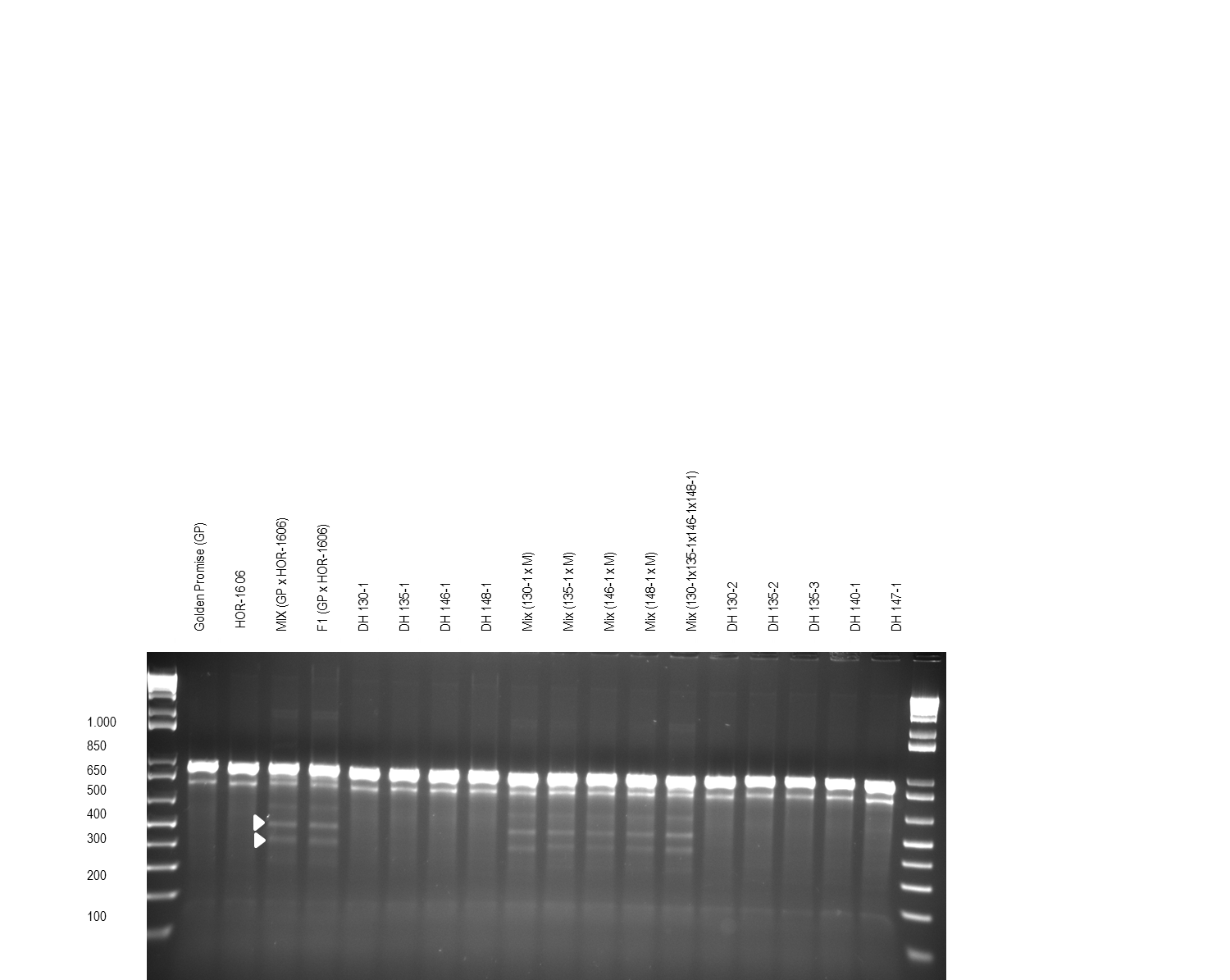


B.

Primer pair: nbs3-rdg2a_F2_R2


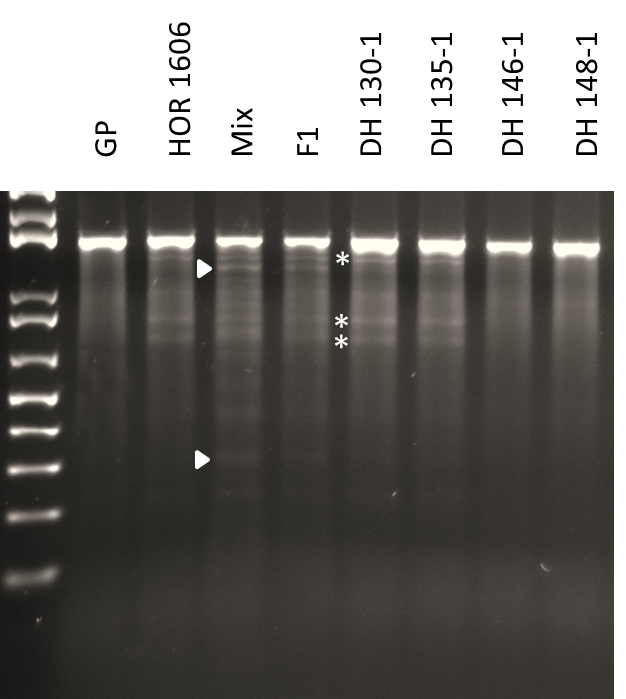


C. Primer pair Kap1_c1


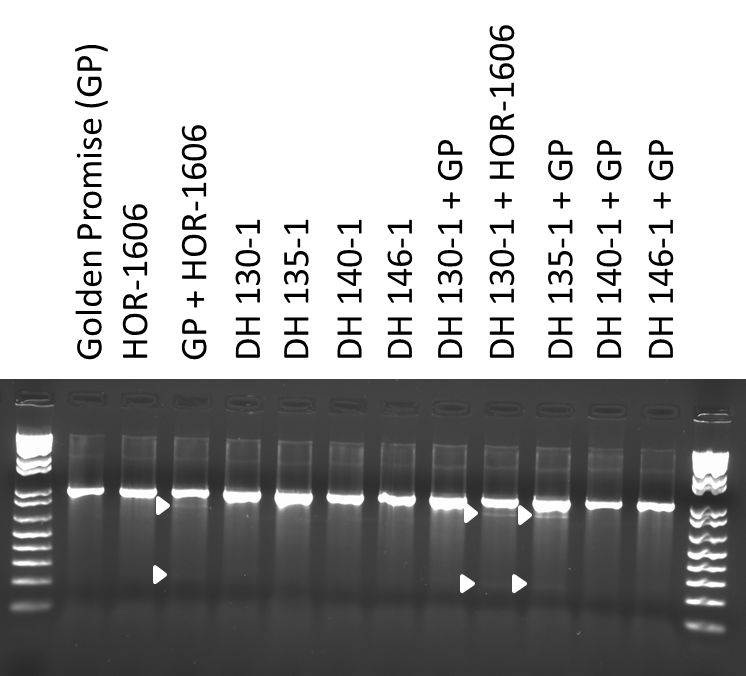


D. Primer pair Kap1_d1


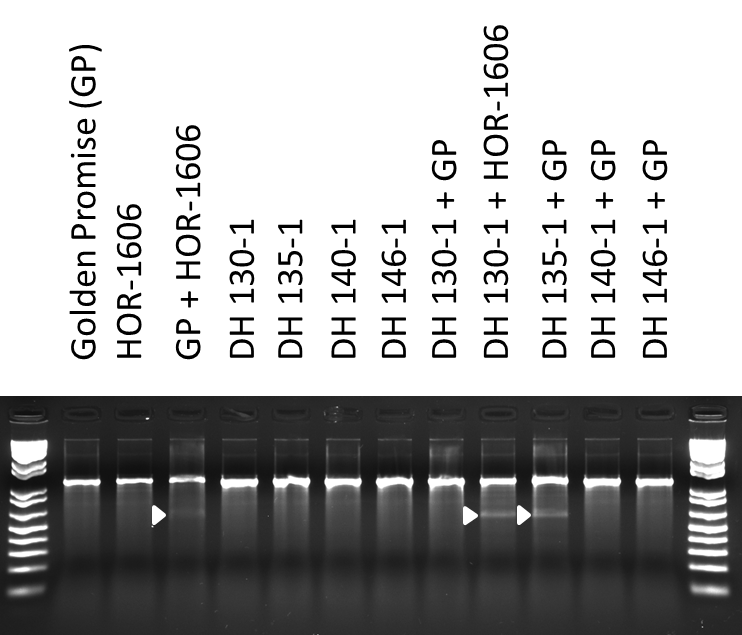


E. Primer pair Kap1_e1


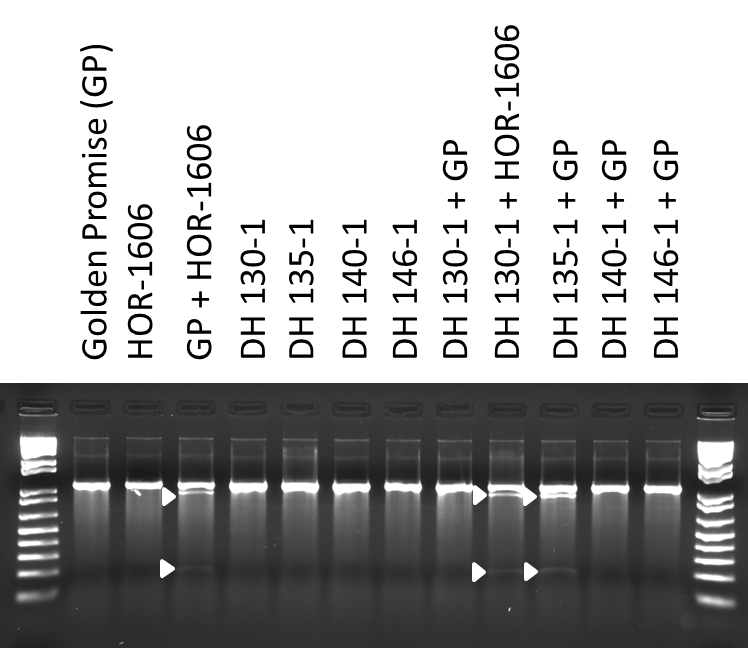


F. Rapid screening of primer pairs with a melting temperature of 65⁰C


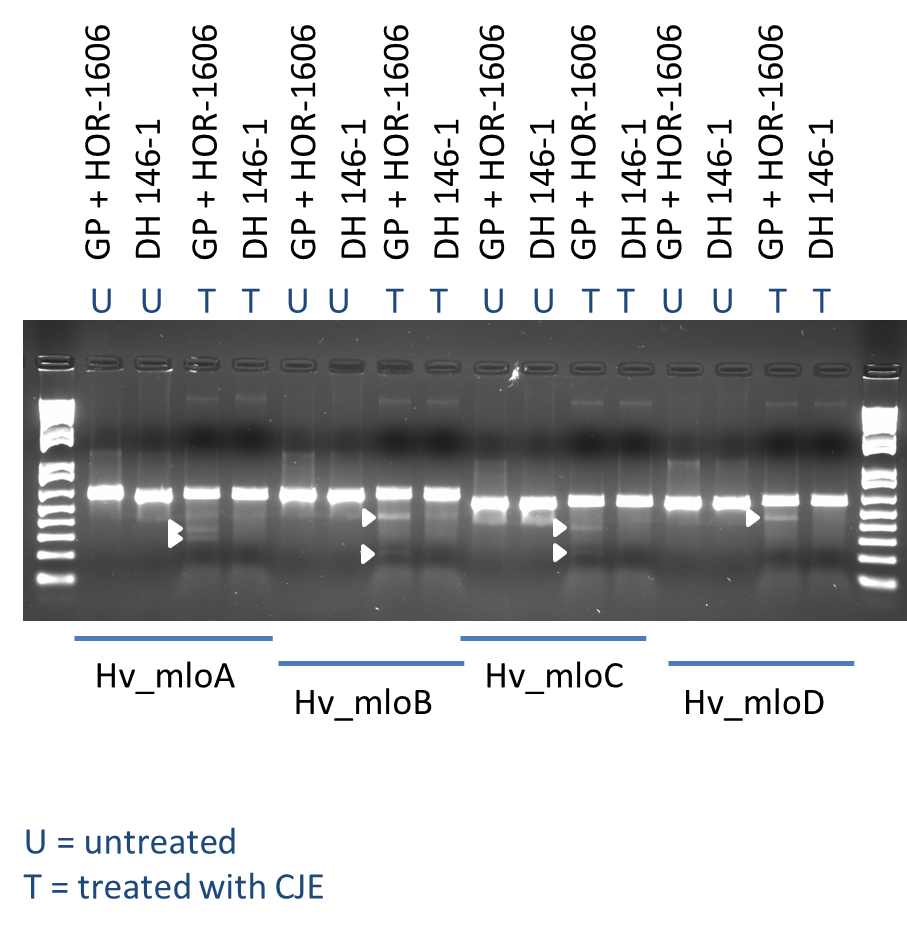


G. Primer pairs FVHox1_F1&R1


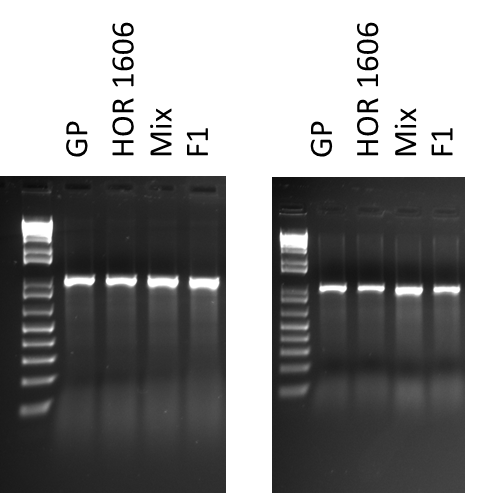

Supplement: Additional file 1 — Agarose gel images of primers evaluated for DH screening by enzymatic mismatch cleavage. Screening with primer pairs HV_Mlo9 (A), and nbs3_rdg2a (B), resulted in the production of cleaved products (marked by arrow heads) in mixed parental and F1 DNA samples indicating suitability for screening doubled haploid plants (lanes marked DH). Primers for nbs3_rdg2a, like those for nbs_rdg2a (Figure 2) produce banding in HOR1606 samples (marked by *), allowing assignment of parental origin in doubled haploid plants without the need to mix DNA from parental genotypes. Primer pairs with a melting temperature of 60°C are also suitable for DH screening (C, D, E). Rapid testing of primer pairs is accomplished by screening by comparing untreated PCR products with those treated with CJE (F). Primer pairs where amplification is observed but no polymorphisms detected in mixed parental or F1 DNA samples are not used for screening putatively doubled haploid material (G, and not shown). [file 1746-4811-9-43-S1.doc]
