# Supplementary material for: Validation of doubled haploid plants by enzymatic mismatch cleavage
Source: Plant Methods. 2013 Nov 13;9:43. doi: 10.1186/1746-4811-9-43 (PMC3831592; doi:10.1186/1746-4811-9-43)

Additional file 2: Agarose gel images of SSR markers for doubled haploid screening. Pilot tests were performed to identify primers showing polymorphism between parental Golden Promise (GP) and HOR 1606 lines. No DNA controls and an additional parent, HOR 2444, were included in each trial. Primer names are included below the image. Examples include weakly amplifying primers (cnl34), primers where no polymorphisms are observed (cnl73, cnl31, cnl130), where mis-amplification occurred in one of the parents (cnl151) and where a size polymorphism could be detected between GP and HOR 1606 (cnl146). Further optimization was performed with cnl146 by reducing extension time from 2 minutes to 30 seconds (not shown).


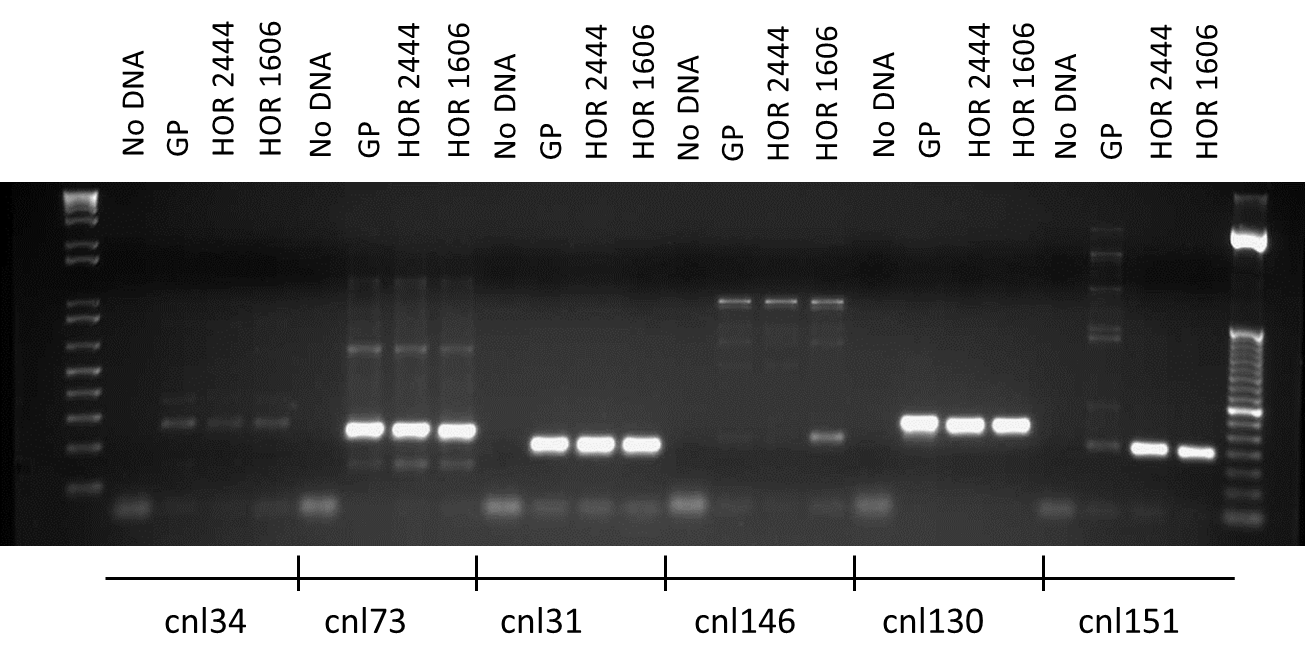

Supplement: Additional file 2 — Agarose gel images of SSR markers for doubled haploid screening. Pilot tests were performed to identify primers showing polymorphism between parental Golden Promise (GP) and HOR1606 lines. No DNA controls and an additional parent, HOR2444, were included in each trial. Primer names are included below the image. Examples include weakly amplifying primers (cnl34), primers where no polymorphisms are observed (cnl73, cnl31, cnl130), where mis-amplification occurred in one of the parents (cnl151) and where a size polymorphism could be detected between GP and HOR1606 (cnl146). Further optimization was performed with cnl146 by reducing extension time from 2 minutes to 30 seconds (not shown). [file 1746-4811-9-43-S2.doc]
